# Association of Selenium Intake and Development of Depression in Brazilian Farmers

**DOI:** 10.3389/fnut.2021.671377

**Published:** 2021-05-20

**Authors:** Tatiana Lourençoni Ferreira de Almeida, Glenda Blaser Petarli, Monica Cattafesta, Eliana Zandonade, Olivia Maria de Paula Alves Bezerra, Kelly Guimães Tristão, Luciane Bresciani Salaroli

**Affiliations:** ^1^Graduate Program in Nutrition and Health, Federal University of Espírito Santo, Vitória, Brazil; ^2^Clinical Nutrition Unit of the Cassiano Antonio Moraes Hospital (HUCAM) of the Federal University of Espírito Santo, Vitória, Brazil; ^3^Postgraduate Program in Public Health, Federal University of Espírito Santo, Vitória, Brazil; ^4^Department of Statistics and the Graduate Program in Collective Health at the Federal University of Espírito Santo, Vitória, Brazil; ^5^School of Medicine, Federal University of Ouro Preto, Ouro Preto, Brazil; ^6^Analytical Psychology Center of Espírito Santo, Vitória, Brazil; ^7^Department of Integrated Health Education, Postgraduate Program in Nutrition and Health, Postgraduate Program in Collective Health of the Federal University of Espírito Santo, Vitória, Brazil

**Keywords:** rural worker, rural population, food consumption, micronutrient, selenium, depression, public health

## Abstract

**Introduction:** Depression and deficiency in the consumption of micronutrients are a public health problem, especially in the rural population. The deficiency in selenium consumption affects mental health, contributing to the development of major depressive disorders. Thus, this study aimed to evaluate selenium intake and its association with depressive symptoms in farmers in southeastern Brazil.

**Material and Methods:** Epidemiological, cross-sectional, and analytical study with 736 farmers aged between 18 and 59. A semistructured questionnaire was used to collect sociodemographic, lifestyle and clinical condition data. For evaluation of food intake, three 24-h recalls were applied, and for identification of depressive episodes, the Mini-International Neuropsychiatric Interview was conducted.

**Results:** A total of 16.1% (*n* = 119) of the farmers presented symptoms of major depressive episodes, 5.8% (*n* = 43) presented symptoms of current depressive episodes, and 10.3% (*n* = 76) presented symptoms of recurrent major depressive episodes. Sociodemographic factors associated with depression were gender (*p* < 0.001), marital status (*p* = 0.004), and socioeconomic class (*p* = 0.015). The consumption of high doses of selenium was associated with a reduction of ~54% in the chances of occurrence of depression (OR = 0.461; 95% CI = 0.236–0.901).

**Conclusion:** High selenium intake is associated with a lower prevalence of depression even after adjusting for sociodemographic variables, lifestyle, and pesticide intoxication. The findings of this study contributed to highlighting the high prevalence of depression in rural areas and its relationship with selenium intake.

## Introduction

Depression is a common mental disorder all over the world and the leading cause of inability (1)^1^. It contributes considerably to the global burden of disease and leads to loss of productivity and increased time away from work (2). It is estimated that 322 million people worldwide have this disease. Globally, depressive disorder is ranked as the largest contributor to disability-adjusted life-years (DALYs) with 7.5% of years lived with disability (YLD) (3)^2^. The Global Burden of Disease Study, a systematic analysis of the global burden of disease, examined 369 diseases and injuries in 204 countries and territories between 1990 and 2019, identifying the 10 most important factors that increase the burden of disease—among them, depressive disorders that are common from adolescence to old age (4)^3^.

One of the major contributing factors to the progressive increase of chronic non-communicable diseases such as depression is an unhealthy lifestyle, including an inadequate diet (5). There is evidence of the protective effect of certain dietary patterns and nutrients on depression occurrence. Among the mechanisms involved in this relationship are a decrease in oxidative stress, reduction in inflammatory markers, improvement in endothelial function, and alteration in the synthesis and function of serotonin (6). Additionally, it is known that long-term exposure to low levels of some nutrients, like selenium, may affect brain function, such as cognitive function and mood (7).

Selenium is an essential nutrient necessary for the optimal functioning of several selenoproteins. The role of selenium in the development of depression seems to be related to its ability to reduce oxidative stress and inflammatory markers, improving endothelial function and altering the synthesis and functioning of serotonin (6, 8, 9). The modulatory effects of selenium on thyroid metabolism also seem to influence an individual's susceptibility to developing depression, as well as the action of selenoproteins in the dopaminergic, serotonergic and noradrenergic systems (10). A study involving more than 14,000 individuals showed that participants who met the RDA (Recommended Dietary Allowance) for selenium had significantly lower chances of depression (OR: 0.52; 95% CI: 0.39, 0.71) (11). Selenium concentrations were also significantly lower in patients with major depressive disorder compared to controls in Bangladesh (*p* < 0.05) (12). In rats, higher levels of selenium have been shown to be protective against the development of depressive symptoms in response to stress (13). Despite this, the evidence is still contradictory (9–11, 14, 15).

In Brazil, few studies evaluate the consumption of micronutrients in the population (7). Thus, the aim of this study was to evaluate selenium intake and its association with depressive symptoms in Brazilian farmers and to analyze whether farmers with highest quartile selenium intake are less likely to develop depressive symptoms when compared to farmers with the lower quartile of selenium intake.

## Materials and Methods

### Study Design

This was a quantitative analytical epidemiological study derived from a larger population-based project entitled “Health status and associated factors: A study in farmers in Espírito Santo—AgroSaúdES.” The target population of this research was farmers of both sexes, aged between 18 and 59 years, working in the municipality of Santa Maria de Jetibá - ES, Brazil. The inclusion criteria were that farmers had to be between 18 and 59 years old, had to not be pregnant, had to have agriculture as the main source of income, and had to be in full employment for at least 6 months. Farmers who did not meet the inclusion criteria, did not participate in one of the stages of data collection, or did not sign the informed consent form (ICF) did not participate in the research.

The sample size was calculated by considering an estimated prevalence of depression in rural populations of 5.6% (16), an error rate of 2%, and a confidence interval of 95%, with a minimum required sample size of 468 individuals. However, to improve the representativeness of the sample and the statistical relevance, data from all farmers who participated in the original project and who had data of interest available were used. The sample size was calculated using the EPIDAT program version 3.1. Farmers who met the inclusion criteria were identified based on the data available in the registers of individuals and families filled out by Family Health Strategy teams responsible for covering 100% of the 11 health regions that made up the municipality.

Through these records, we identified 7,287 farmers belonging to a total of 4,018 families who met the inclusion criteria. Participants were selected through stratified draw after considering the number of families per health region and per community health agent (CHA), and the proportionality among the 11 regions and the 80 CHAs. Only one randomly selected individual per family was admitted, thus avoiding the interdependence of information. In case of refusal or non-attendance, a new participant was called from a reserve list of the draw, based on the sex and health unit of origin of the person who dropped out. A total of 806 farmers were invited to compensate for possible losses. Of these, 790 agreed to participate in the research and signed the ICF. Subjects who did not respond to the three 24-h recordings (*n* = 50) and who had attempted suicide by ingesting pesticides (*n* = 4) were excluded from the study, leaving a total of 736 farmers.

### Study Environment

The study was conducted among farmers in the municipality of Santa Maria de Jetibá, a mountainous region of the state of Espírito Santo, Brazil. The study was conducted here because it is the largest producer of horticultural products in Espírito Santo (17).

The population is predominantly rural and carries out family farming as its main economic activity. Its agricultural practices are characterized by the predominance of polyculture and a low degree of mechanization. The population follows a contemporary food pattern characterized by a traditional Brazilian pattern and a local yet industrialized pattern, indicating that the farmers follow a diet with ultra-processed products and low fruit consumption, and that they have habits characteristic of more urbanized rural regions (18). Ultraprocessed foods are characterized by high energy, low fiber, and microscopic minerals and high added or free sugars, sodium, saturated fats, and chemical food additives (19).

### Ethics

The Research Ethics Committee of the Federal University of Espírito Santo—Opinion No. 2091172 (CAAE 52839116.3.0000.5060) approved the project and complied with the ethical rules governing research involving human beings. All participants who agreed to participate in the research signed the Free and Informed Consent Form. The research was conducted in partnership with the Municipal Health Secretariat and the Rural Workers Union of the municipality of Santa Maria de Jetibá.

### Data Collection

Data were collected from December 2016 to April 2017. It should be noted that, given the predominance of polyculture and the production of short-cycle foods such as vegetables (20), no major differences were identified between the months selected for data collection and the other months of the year with respect to agricultural practices, including those related to pesticide use. The selected farmers were grouped by region of residence and invited by the CHAs to come to the basic health unit on a predefined day and time for data collection. Data collection was structured as follows: after signing the ICF, the farmer answered the semi-structured questionnaire to provide socioeconomic and occupational, lifestyle habit, risk perception, behavior adopted during pesticide handling, and self-reported disease and symptom data. The data collection team was composed of five trained permanent members: one PhD student, two master's students, one graduate student, and one undergraduate student. To minimize inter-observer variability, the researchers remained in the same positions from the beginning to the end of the data collection process, and four interviewers administered the questionnaires.

### Selenium Intake Analysis

Selenium intake was assessed using three 24-h recordings (24 h). Given the high variability in nutrient intake on different days, two 24 h recall schedules were applied to 2 days of the week and one 24 h recall schedule on the weekend was added to be more representative of the usual intake since there are significant variations between these days. From the results of the three recall schedules, an average was made that depicts the usual intake of the study population. This analysis methodology is in accordance with the protocol widely used in the literature of de area in several studies that evaluate the food consumption of populations (21, 22). The first recall was applied during the interview, in which individuals reported all the food and beverages consumed, including the respective amounts and portion sizes consumed in the last 24 h. The second recall was performed within 7 days after the first 24 h data collection, and the third recall was performed during the return visit, which took place 8–15 days after the first contact with the interviewee. To ensure greater accuracy of the portions eaten, photo albums were used to facilitate the identification and quantification of the consumed items. In the case of processed products, the brands, type, and quantity of the product were recorded. The nutritional composition analysis of the 24 h was conducted later by means of the AvaNutri 4.1 program and the Brazilian Table of Food Composition (TACO) (23). The typical foods of the region were registered in the software according to manufacturer information or standardized recipes.

After the food and nutrient intake was registered, it was observed that none of the participants had energy consumption equal to or <500 kcal and more than 6,000 kcal (22). Thus, they presented values compatible with the usual food consumption patterns, and it was not necessary to exclude any individual from the analyses. After obtaining the values of each 24 h, deattenuation analysis was conducted using PC-SIDE software (Department of Statistics, Iowa State University, Iowa, United States). Energy adjustment was also performed using the residual method, which, according to Willett, corrects nutrient estimates by total energy intake, thus providing the energy-adjusted selenium value (22). Variation of selenium intake was expressed in micrograms/day (μg/day) and in quartiles, allowing risk trend analysis according to the degree of exposure and the differences between extreme intake concentrations which allows comparison of risk between lower and higher quartiles of food intake (24). Selenium intake in the evaluated sample ranged between 28.15 and 146.83 μg/day. No farmer in the study had a consumption higher than the UL established for this micronutrient. Thus, the first quartile contained individuals with a daily selenium intake of 66.66 μg, the second quartile contained those who had a daily selenium intake of 66.67–80.37 μg, the third quartile contained those who had a daily selenium intake of 80.38–95.25 μg, and the fourth quartile contained those who had a daily selenium intake of above 95.26 μg.

### Analysis of Depressive Symptoms

To evaluate the symptoms of depression, we used the Major Depressive Episodic Module of the Brazilian version of the Mini-International Neuropsychiatric Interview (MINI), which is organized into independent diagnostic modules. It is a brief standardized diagnostic interview with good sensitivity and specificity for use in clinical practice and research (25), which aims to diagnose interviewees in a way that is compatible with the criteria of the Diagnostic and Statistical Manual of Mental Disorders (26) and the International Classification of Diseases (27). The version used in the present study corresponded to MINI 5.0 in Portuguese (25). The individuals were classified into two categories: “without depression episode” or “with depression episode.” The latter category contained two subcategories, “current depressive episode” or “recurrent depressive episode,” based on the version MINI 5.5 mentioned above. According to literature data, the results concerning the reliability and validity of this instrument were globally satisfactory (25).

### Independent Variables

The independent variables were subdivided into sociodemographic variables, lifestyles, clinical conditions, and selenium consumption. Sociodemographic variables included gender, age group (“up to 29 years,” “30–39 years,” “40 years or older”), race/color (“white” and “non-White”), education (“<4 years,” “4–8 years,” “more than 8 years”), marital status (“single,” “married/living with a partner,” and “separated/divorced/widowed”), and socioeconomic class (“class A or B,” “class C,” and “class D or E”). National studies use this classification. Socioeconomic classes are estimated according to the purchasing power of individuals and families, allowing the estimation of the average monthly gross family income (A: ~R$ 11,037.00; B: ~R$ 6006.00; C: ~R$ 1,865.00; D/E: ~R$ 895.00) (28)^4^. For the lifestyle variable, the consumption of alcohol (“consumes” and “does not consume”) and tobacco (“smoker or former smoker” and “non-smoker”) was analyzed. The clinical condition variable analyzed the diagnosis of pesticide intoxication made by a health professional and involved the question, “Has a doctor or other health professional ever diagnosed you with pesticide intoxication?” The responses were categorized as “yes” and “no.”

### Statistical Analysis

Statistical analyses and the interpretation of the results followed the consistency of the theoretical model used to investigate the relationship between selenium consumption and depression. The absolute and relative frequencies of the independent variables were calculated according to the presence or absence of depression. To evaluate the qualitative variables and their associations, the Chi-squared test of association was used. Variables with *p*-value < 5 (5% significance level) in this test were included in the binary logistic regression analysis as the following adjustment factors: sociodemographic variables (gender, marital status, socioeconomic class); lifestyle (alcohol consumption); clinical condition (pesticide intoxication), and selenium consumption. Pesticide poisoning was included as an adjustment variable as it has often been associated with the occurrence of depression (29–31).

The results were expressed as odds ratios (OR) along with the respective confidence intervals. The quality of the model was assessed using the Hosmer-Lemeshow test. All data were organized and analyzed in IBM SPSS® version 22.0 software.

## Results

In this study, 378 (51.33%) farmers were male, and 358 (48.64%) were female. The majority (535, 73.1%) were above 30 years old. A total of 497 (67.53%) of them had an education level below 4 years of study, 666 (90.49%) were of White race/color, and 680 (92.39%) belonged to classes C, D, and E.

**Figure 1:**
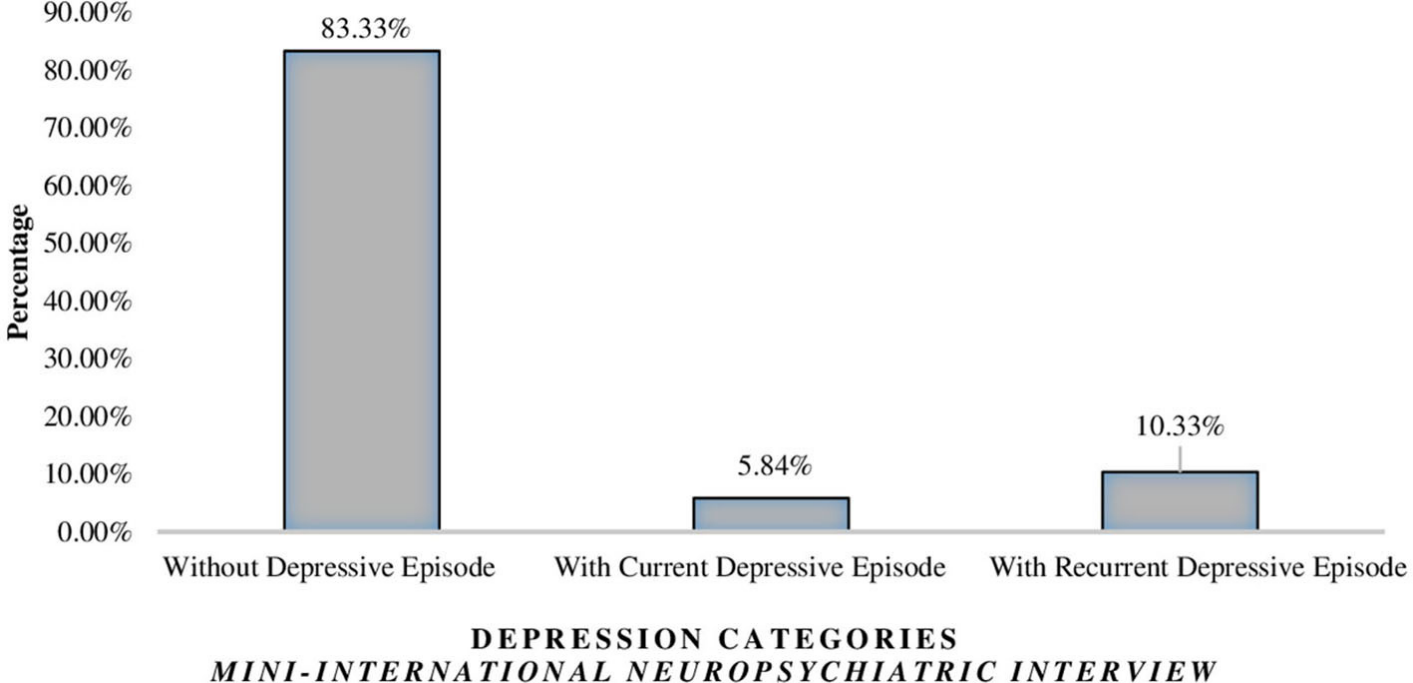
Prevalence of major depressive episodes in farmers according to the MINI scale version 5.0.

Of the 736 farmers who were assessed, 617 (83.83%) were classified as without depressive episodes. The prevalence of major depressive episode was 16.17% (*n* = 119). Subdivided by recurrence, 43 (5.84%) participants had current major depressive episode, and 76 (10.33%) had “recurrent major depressive episode” (Figure 1).

The variables associated with depression that were entered into the logistic regression model as adjustment variables were as follows: sex (*p* < 0.01), marital status (*p* = 0.004), socioeconomic class (*p* = 0.015), alcohol consumption (*p* = 0.002), and pesticide intoxication (*p* = 0.001). Most subjects did not consume alcohol (56.66, *n* = 417), were not smokers (85.33 %, *n* = 628), and had not been diagnosed with pesticide intoxication (93.2%, *n* = 686) (Table 1).

**Table 1:** Prevalence of depression according to sociodemographic characteristics, lifestyles, and pesticide intoxication among Brazilian farmers.

	**Depression**						
**Variables**	**Total**		**No**		**Yes**		***P*-value**
	***n***	**%**	***n***	**%**	***n***	**%**	
**Sex**							
Male	378	51.36	336	88.90	42	11.10	**<0.001**
Female	358	48.64	281	78.50	77	21.50	
**Age group**							
Up to 29 years	201	27.31	171	85.10	30	14.90	0.753
30–39 years	217	29.48	183	84.30	34	15.70	
40 or more	318	43.21	263	82.70	55	17.30	
**Marital status**							
Single	56	7.61	52	92.90	4	7.10	**0.004**
Married/living with partner	635	86.28	534	84.10	101	15.90	
Separated/divorced/widowed	45	6.11	31	68.90	14	31.10	
**Education**							
<4 years	497	67.53	418	84.10	79	15.90	0.713
4–8 years	161	21.88	132	82.00	29	18.00	
More than 8 years	78	10.59	67	85.90	11	14.10	
**Race/color**							
White	666	90.49	561	84.20	105	15.80	0.36
Non-white	70	9.51	56	80.00	14	20.00	
**Socioeconomic class**							
Class A or B	56	7.61	52	92.90	4	7.10	**0.015**
Class C	375	50.95	322	85.90	53	14.10	
Class D or E	305	41.44	243	79.70	62	20.30	
**Alcohol consumption**							
Does not consume	417	56.66	334	80.10	83	19.90	**0.002**
Consumes	319	43.34	283	88.70	36	11.30	
**Smoking**							
Non-smoker	628	85.33	529	84.20	99	15.80	0.473
Current and past smoker	108	14.67	88	81.50	20	18.50	
**Intoxication by agrochemicals**							
Yes	49	6.65	33	67.30	16	32.70	**0.001**
No	686	93.2	583	85	103	15.00	

*Chi-squared test. In bold: Statistically significant values (p < 0.05)*.

When the association of selenium consumption with depression was analyzed, 33 individuals (17.8%) in the lowest quartile of consumption showed depression, whereas 17 individuals (9.2%) in the highest quartile of consumption showed this morbidity (Table 2).

**Table 2:** Prevalence of depression according to quartile of selenium consumption of Brazilian farmers.

	**Depression**						
**Variable**	**Total**		**No**		**Yes**		***P*-value**
	***n***	**%**	***n***	**%**	***n***	**%**	
**Selenium consumption**							
Quartile 1 (≤66.66 μg)	185	25.13	152	82.20	33	17.80	**0.032**
Quartile 2 (66.67–80.37 μg)	184	25	150	81.50	34	18.50	
Quartile 3 (80.38–95.25 μg)	183	24.87	148	80.90	35	19.10	
Quartile 4 (>95.26 μg)	184	25	167	90.80	17	9.20	

*Chi-squared test. In bold: Statistically significant values (p < 0.05)*.

Below are the binary logistic regression data between depression and selenium consumption adjusted for sociodemographic variables (gender, marital status, economic class), lifestyle (alcohol consumption), and pesticide intoxication (Table 3).

**Table 3:** Binary logistic regression [OR (95% CI)] between depression and highest quartile of selenium consumption, gender, marital status, socioeconomic class, alcohol consumption, and pesticide poisoning in Brazilian farmers.

	**OR crude**		OR adjusted^*^	
	**OR**	**CI 95%**	**OR**	**CI 95%**
**Depression**				
No	Reference^**^		Reference^**^	
Yes	0.469	(0.251–0.876)	0.461	(0.236–0.901)

* *Adjusted for gender, marital status, socioeconomic class, alcohol consumption, and pesticide poisoning*.

** *Statistically significant values (p < 0.05*). *Hosmer-Lemeshow = 0.795*.

The comparison of the first quartile (lowest consumption) with the fourth quartile (highest consumption) showed that the prevalence of depression was significantly lower in farmers with higher consumption of selenium (Q4), even after adjusting for sociodemographic variables, lifestyle and pesticide poisoning (OR = 0.461; 95% CI = 0.236–0.901) (Table 3).

## Discussion

This is the first Brazilian study to evaluate the relationship between selenium intake and depression in farmers and to contribute to filling this gap in the literature, especially in rural populations. It has been argued that studies on the role of nutrition in psychiatric diseases are essential to expand the role of health professionals with individuals with this morbidity (32). This study is justified by its relevance to public health, especially given the high incidence and prevalence of depression in recent years in the general population, and the scarcity of information regarding mental health in farmers (30, 33)—especially depression (34, 35)—as well as of nutritional and anthropometric assessments of this population (18, 36, 37). The representative and randomly selected sample of this study allowed us to extrapolate the results to the target rural population.

Importantly, the proportion of the global population with depression in 2015 was estimated to be 4.4%, and the estimated total number of people in the world living with depression increased by 18.4% between 2005 and 2015, totaling 322 million people with depression worldwide. The Americas were ranked fourth in the global population with the highest prevalence of depression (15%), followed by the Southwest Asian (27%), Western Pacific (21%), Mediterranean (16%), European (12%), and African (9%) regions (3). In the population studied, there was a high prevalence of depression (16.17%) (35). This data is alarming since the prevalence of depression found in this study is higher when compared to some Brazilian studies. This can be verified in the National Health Survey (30), which indicated the prevalence of depression in Brazilian adults (7.6%), namely, the rural population (5.6%) and Espírito Santo (5.5%). Another study conducted in a rural population in southern Brazil found the prevalence of depression to be 8.1% (38). These data were corroborated by international studies carried out among the rural populations of Lithuania, China, and Canada (7, 33, 39).

Further, the sample studied presented a contemporary food pattern characterized by a “traditional Brazilian pattern,” “local traditional” and “industrialized,” that opts for a diet with ultra-processed products and low fruit consumption—habits characteristic of more urbanized rural regions (18).

This eating behavior results in inadequate micronutrient intake (40), worse health outcomes, and overall poor diet quality (41), and may be directly associated with depressive feelings (29) and poor mental health. These data confirm the trend indicated by the literature of increasing adherence of residents of rural areas to the dietary patterns of urban areas (42). The process of urbanization and globalization have led to the mitigation of regional differences and to the increase of integration, exchange of information, and access to variable and healthy foods (43)^5^.

The sociodemographic, lifestyle, and work variables associated with depression were used in this study as adjustment variables to corroborate the data in the literature.

The results demonstrated the association between selenium consumption and the prevalence of depression in rural areas. Farmers in the highest quartile of selenium consumption were 54% less likely to experience depression compared to the lower quartile of consumption. Corroborating these findings, a cross-sectional study with 14,834 adults from the National Health and Nutrition Examination Survey (NHANES) 2009–2014 identified Odds ratios of 0.46 (0.32–0.67) for the highest quartile vs. the lowest quartile of selenium intake (10), values very close to those found in the present investigation. Randomized clinical trials found improvement in mood (44, 45) and improvement in post-partum depression (46) with selenium supplementation in adult populations. In the United States, a rural health study supported the link between exposure to selenium in groundwater and decreased symptoms of depression (47). In the MASHAD study, selenium intake was negatively associated with the relative risk of a high depression score using the Beck scale (48). In contrast, the results of two cross-sectional studies conducted among a geriatric population in rural China (7), as well as a population of patients on hemodialysis (49), found no significant association between selenium levels and depression scores after controlling for chronic kidney disease and cognitive function. Research with the American adult population also found no association between these variables (15).

It should be noted that excessive selenium consumption also seems to be related to a higher risk of developing depression, corroborating the hypothesis of a U-association between these two variables (15). Considering that, among the evaluated farmers, no individual exceeded the intake above the UL established for selenium (400 μg/day), the evaluation of the effects of excessive consumption of this nutrient cannot be performed in this study. The average selenium intake among the evaluated farmers was 81.27 μg/day, higher than that found in other countries such as New Zealand (50) and Poland (51). Only 8.8% (*n* = 65) of farmers had lower consumption than the Recommended Dietary Allowances (RDAs) of 55 μg/day established for this nutrient. These results are possibly due to the high consumption of meat, eggs and minimally processed food sources identified in this population (18, 52). The high intake of this micronutrient in the Brazilian population has also been reported by other studies (53).

A case-control study with 1,494 women aged 20–89 years reported that dietary intake of lower selenium (<8.9 μg/day) was associated with an increased risk of developing major depressive disorder (14). In another US study of 30 men on a low or high selenium diet (32.6 μg vs. 226.5 μg/day), mood deteriorated with the poor diet and improved with the rich diet (54). A study in post-partum mothers found that depressive symptoms were lower around serum selenium concentrations of 82–85 mg/L. Below 82 mg/L, depressive symptoms began to increase, culminating in the highest depressive symptoms for participants in the lowest decile of serum selenium (approximate serum selenium concentration of 62 mg/L). Concentrations >110 mg/L have also been associated with an increase in depressive symptoms (50).

It is important to highlight that the understanding of the association of selenium consumption and depression found is due to the essential role of selenium in health, mood, and the physiology of depression (6, 10, 55).

According to WHO (56)^6^. mental disorders result from many factors and have their physical basis in the brain. This is an organ predisposed to oxidative-nitrosative stress. If its antioxidant defenses do not react adequately to radical damage, the neurons may undergo microalteration, microdysfunction, and degeneration, contributing to the pathogenesis of depressive disorders (57). Additionally, depression has been recognized as an inflammatory disorder accompanied by an accumulation of reactive oxygen species that overwhelm the physiological processes of the individual. This suggests that depression is a disease belonging to the (neuro)degenerative disorder spectrum (58). There are several possible physiological hypotheses about the effects of selenium on mood enhancement, including its role in maintaining metabolic, oxidative, and central nervous system function, as well as the potential underlying mechanisms between low serum selenium levels and the development of depression, such as dysregulation of thyroid function and oxidative and inflammatory pathways (10). However, studies examining the relationship between depression and selenium are largely inconclusive (10), and further research is needed to clarify selenium's actions on the other physiological mechanisms of depression.

The implications of this work are that, despite working in rural areas, the food intake of the farmer population is inadequate and needs to be better observed. Broader analyses of food intake are needed to verify the inadequacy of other micronutrients. Dietary adequacy is necessary because depression may be related to inadequate intake of other micronutrients (10, 59, 60) and the lack of important nutrients in a diet creates risk for many non-transmissible chronic diseases (49)^7^. Considering all the economic, social, and health implications resulting from the inadequacy of food intake and depression, such as a loss of productivity, an increase of sick leaves (2), and the disease burden itself (1), it is necessary to reflect on strategies of confrontation and prevention of this problem to improve workers' health.

The implementation of food education programs is essential to strengthen the health surveillance system and promote an adequate and healthy diet for farmers. Furthermore, it is essential to ensure access to mental health care services in primary care to support the prevention and treatment of depression in rural areas from the comprehensive viewpoint of the farmer, because few effective interventions exist that can reduce the vulnerability of rural workers (55). The main finding of the study was the height selenium intake was associated with reduction in the prevalence of depression in Brazilian farmers. The result contribute to the limited literature on mental health and selenium consumption in rural workers by showing evidence of an association between high selenium intake and reduced rates depression.

### Final Consideration

Higher selenium intake was associated with a lower prevalence of depression in the rural Brazilian population. Actions to promote adequate nutrition are important to reduce the vulnerability of rural workers to depressive disorders.

To better understand the influence of selenium consumption on the development of depression, studies with a longitudinal design and the inclusion of biochemical measures for selenium measurement are necessary.

### Strengths and Limitations

It is important to mention the limitations of this study. Its cross-sectional nature created limitations inherent to this type of epidemiological study. It made reverse causality possible, given that the association between the variables was synchronous. Therefore, it is not possible to infer causality between the variables evaluated. However, the diagnostic scale adopted investigated the symptomatology of depressive disorders in the last 2 weeks, which favored temporal analysis of the observations. Another possible limitation to consider is the information retrieval bias related to recent memory and the inaccurate estimation of ingested portion sizes, amounts, and frequencies in assessing the nutritional status of the participants (61). To minimize the effect of these biases and to ensure greater accuracy of the ingested portions, photographic albums were used to facilitate the identification and quantification of the consumed items, as well as—in the case of industrialized products—to register the brands, types, and quantities of the products. There was also the possibility of respondent bias, in which the individual tends to overestimate the consumption of healthy foods and underestimate that of unhealthy foods because of the stereotype of a healthy lifestyle based on agriculture and health diet based on natural foods (62). However, the occurrence of this bias was unlikely because the study population followed a contemporary dietary pattern, as already described.

Biochemical measurements of selenium were not used. The use of dietary data alone may limit the assessment of selenium intake. However, in an attempt to minimize this weakness, several precautions were adopted, including methodological rigor in obtaining information regarding farmers' food intake, to ensure that, in fact, it could reflect the actual consumption of the assessed population. The calculation of the average consumption was performed based on the information obtained after the application of three 24-h reminders (including weekdays and weekends), expanding the capacity of this instrument to detect variations in consumption between the days of the week. In addition, the average selenium intake value was obtained by calculating the attenuated and energy-adjusted average, as a way of reflecting the usual consumption of the evaluated sample. Photo albums were also used to facilitate the identification and quantification of the items consumed. In addition, data collection was performed by a fixed team of trained researchers in order to reduce the variability between observers. It is also noteworthy that the assessment of the nutritional composition of the foods identified in the 24-h recalls was carried out using a Brazilian food composition table (TACO) (23) as a way to more accurately reflect the content of the nutrient in the food. The selection of a large and representative sample, at random, also attributed greater robustness to the data obtained.

Despite these limitations, we highlight the unprecedented character of the study in the Brazilian literature in relation to the target population involved, the rigorous methodology of recruiting participants and assessing food consumption, the large sample size, the adoption of a validated diagnostic scale to obtain the results of depression and the inclusion, in statistical analysis, of other variables with potential influence on the development of depression.

## Data Availability Statement

The original contributions presented in the study are included in the article/supplementary material, further inquiries can be directed to the corresponding author.

## Ethics Statement

The studies involving human participants were reviewed and approved by Ethics Committee on research with human beings - CEP/UFES: n° 2091172 (CAAE - Certificate of Presentation of Ethical Appreciation - 52839116.3.0000.5060). The patients/participants provided their written informed consent to participate in this study.

## Author Contributions

GP, LS, TF, and MC: conception/design of the work. EZ, GP, LS, MC, and OB: data acquisition. GP, KT, LS, MC, and TF: data analysis/interpretation. TF: drafting of the manuscript. EZ, GP, KT, MC, LS, TF, and OB: substantial revision of the manuscript. All authors: approval of the submitted version and taking of personal responsibility for any part of the work.

## Conflict of Interest

The authors declare that the research was conducted in the absence of any commercial or financial relationships that could be construed as a potential conflict of interest.

## Footnotes

^1^ https://www.paho.org/bra/index.php?option=com_content&view=article&id=5635:folha-informativa-depressao&Itemid=1095

^2^ https://apps.who.int/iris/bitstream/handle/10665/254610/WHO-MSD-MER-2017.2-eng.pdf;jsessionid=129DFEF24B5BE7B74114594D24DCA6D4?sequence=1

^3^ https://www.thelancet.com/journals/lancet/article/PIIS0140-6736(20)30925-9/fulltext

^4^ http://www.abep.org/criterio-brasil

^5^ https://biblioteca.ibge.gov.br/index.php/biblioteca-catalogo?view=detalhes&id=291110

^6^ http://www.who.int/whr/2001/en/whr01_ch1_po.pdf

^7^ https://biblioteca.ibge.gov.br/visualizacao/livros/liv50063.pdf
