# Corrigendum: Association of Selenium Intake and Development of Depression in Brazilian Farmers

**DOI:** 10.3389/fnut.2021.756637

**Published:** 2021-08-26

**Authors:** Tatiana Lourençoni Ferreira de Almeida, Glenda Blaser Petarli, Monica Cattafesta, Eliana Zandonade, Olivia Maria de Paula Alves Bezerra, Kelly Guimarães Tristão, Luciane Bresciani Salaroli

**Affiliations:** ^1^Graduate Program in Nutrition and Health, Federal University of Espírito Santo, Vitória, Brazil; ^2^Clinical Nutrition Unit of the Cassiano Antonio Moraes Hospital (HUCAM) of the Federal University of Espírito Santo, Vitória, Brazil; ^3^Postgraduate Program in Public Health, Federal University of Espírito Santo, Vitória, Brazil; ^4^Department of Statistics and the Graduate Program in Collective Health at the Federal University of Espírito Santo, Vitória, Brazil; ^5^School of Medicine, Federal University of Ouro Preto, Ouro Preto, Brazil; ^6^Analytical Psychology Center of Espírito Santo, Vitória, Brazil; ^7^Department of Integrated Health Education, Postgraduate Program in Nutrition and Health, Postgraduate Program in Collective Health of the Federal University of Espírito Santo, Vitória, Brazil

**Keywords:** rural worker, rural population, food consumption, micronutrient, selenium, depression, public health

In the original article, there was an error. There is a sentence in the “discussion” topic in Portuguese language: INCOERENTE COM O OBJETIVO DO ARTIGO? This sentence should have been deleted.

A correction was made to exclude this sentence in Portuguese in the “discussion” section, in the fourth paragraph.

The authors apologize for this error and state that this does not change the scientific conclusions of the article in any way. The original article has been updated.

## Publisher's Note

All claims expressed in this article are solely those of the authors and do not necessarily represent those of their affiliated organizations, or those of the publisher, the editors and the reviewers. Any product that may be evaluated in this article, or claim that may be made by its manufacturer, is not guaranteed or endorsed by the publisher.

